# Scientific success from the perspective of the strength of weak ties

**DOI:** 10.1038/s41598-022-09118-8

**Published:** 2022-03-24

**Authors:** Agata Fronczak, Maciej J. Mrowinski, Piotr Fronczak

**Affiliations:** Faculty of Physics, Warsaw University of Technology, Koszykowa 75, 00-662 Warsaw, Poland

**Keywords:** Complex networks, Computational science

## Abstract

We present the first complete verification of Granovetter’s theory of social networks using a massive dataset, i.e. DBLP computer science bibliography database. For this purpose, we study a coauthorship network, which is considered one of the most important examples that contradicts the universality of this theory. We achieve this goal by rejecting the assumption of the symmetry of social ties. Our approach is grounded in well-established heterogeneous (degree-based) mean-field theory commonly used to study dynamical processes on complex networks. Granovetter’s theory is based on two hypotheses that assign different roles to interpersonal, information-carrying connections. The first hypothesis states that strong ties carrying the majority of interaction events are located mainly within densely connected groups of people. The second hypothesis maintains that these groups are connected by sparse weak ties that are of vital importance for the diffusion of information—individuals who have access to weak ties have an advantage over those who do not. Given the scientific collaboration network, with strength of directed ties measured by the asymmetric fraction of joint publications, we show that scientific success is strongly correlated with the structure of a scientist’s collaboration network. First, among two scientists, with analogous achievements, the one with weaker ties tends to have the higher h-index, and second, teams connected by such ties create more cited publications.

Social networks (SN), representing patterns of human interactions, have been the subject of both empirical and theoretical research since at least the middle of the last century^1^. At the beginning of the twenty-first century, there was a breakthrough in social network analysis (SNA)^2,3^. With the era of widespread digitization, which provided access to huge electronic databases, new empirical methods of SNA have emerged and replaced traditional approaches based on questionnaires and interviews. These new methods, rooted in big data mining, finally allowed for the verification of many well-established theoretical SN ideas, in some cases confirming their validity and in others failing to do so^4^. In this regard, the present status of Granovetter’s weak-tie theory^5,6^ of SN, one of the oldest and most influential theories in sociology, is still vague. There are convincing studies that show the validity of its selected aspects (e.g.,^7–10^), but there are also many that question it (e.g.,^11–13^). Our analysis presented in this paper is unique because, using a massive dataset, not only do we confirm Granovetter’s weak tie theory in its full spectrum but also indicate a possible source of problems related to research questioning its validity.

Granovetter’s theory is based on two hypotheses. The first pertains to the structure of social networks and the second to their dynamics (the way in which the afore-mentioned structure influences the flow of information in the network). It is significant that although most empirical studies have focused on the first hypothesis, far less research has been undertaken to verify the second. One possible reason is that the second hypothesis involves notions relative to the nature and importance of information that are hard to quantify and measure. In this study, we clearly confirm both hypotheses—and Granovetter’s theory in its entirety—in the context of a scientific collaboration network.

The scientific collaboration network^14–18^ is particularly well suited to the overarching goal of this paper (i.e., complete confirmation of Granovetter’s theory) because: (i) connections (ties) between network nodes (scientists) are well defined, and their weight^19^ (strength of ties) is easy to measure (e.g., through joint publications); (ii) scientific publications themselves are also a specific proxy of information flow in the studied network (diffusion of innovations^20^); and (iii) the number of citations is an obvious measure of their significance. Easy access to large datasets is also important, making our conclusions statistically reliable.

The network we investigated has all the features of a complex network^21^. In particular, it shows the scale-free node degree distribution $P(k)\propto k^{-\gamma }$ with the characteristic exponent $\gamma \simeq 2.3$. In the theory of complex networks, this value of $\gamma$ is alarming in the sense that it indicates that the network requires special treatment, including methods of results averaging different to the ones used in homogeneous systems. In relation to Granovetter’s theory, this means that in such networks, basic concepts, such as tie strength and neighbourhood overlap, should be defined in a more careful manner than in homogeneous networks. Their incorrect definition may, instead of confirming the theory, result in its contradiction. In all known empirical studies on Granovetter’s theory, interpersonal ties are assumed to be positive and symmetric. However, it is obvious that social relations do not usually follow this assumption (see, for example, the theory of social balance^22,23^ or the concept of multirelational organization of SN^24,25^). For example, the scientific collaboration between a young scientist and an established one can hardly be called symmetric.

In his original paper^5^, Granovetter treated ties as if they were positive and symmetric, but he also noted that “the comprehensive theory might require discussion of negative and/or asymmetric ties”. We follow this suggestion in this study and reject the assumption about the symmetry of social ties, which is omnipresent in the literature on the subject. The validity of this approach can be explained by intuition trained in the field of complex networks. Granovetter argued that “the degree of overlap of two individuals’ friendship networks varies directly with the strength of their tie to one another”. However, from the theory of complex networks, we know that in social networks with a high degree of heterogeneity (e.g., due to scale-free node degree distribution), the sizes of ego-networks of two connected nodes may differ drastically. Therefore, their common neighbours can be a significant part of the neighbourhood of one node and an insignificant part of the neighbourhood of the other, resulting in a completely different perception of the strength of the link on both ends.

In what follows, we show that the above reasoning, which assumes the asymmetry of tie strength, allows for a quantitative validation of Granovetter’s theory in scientific collaboration networks, that have resisted such verification so far. We use the DBLP Computer Science Bibliography dataset, which includes information on nearly five million computer science papers (i.e., their publication dates, lists of authors and citation records) authored by over four million scientists (see “Data availability” section for more details).

## Results

**Figure 1:**
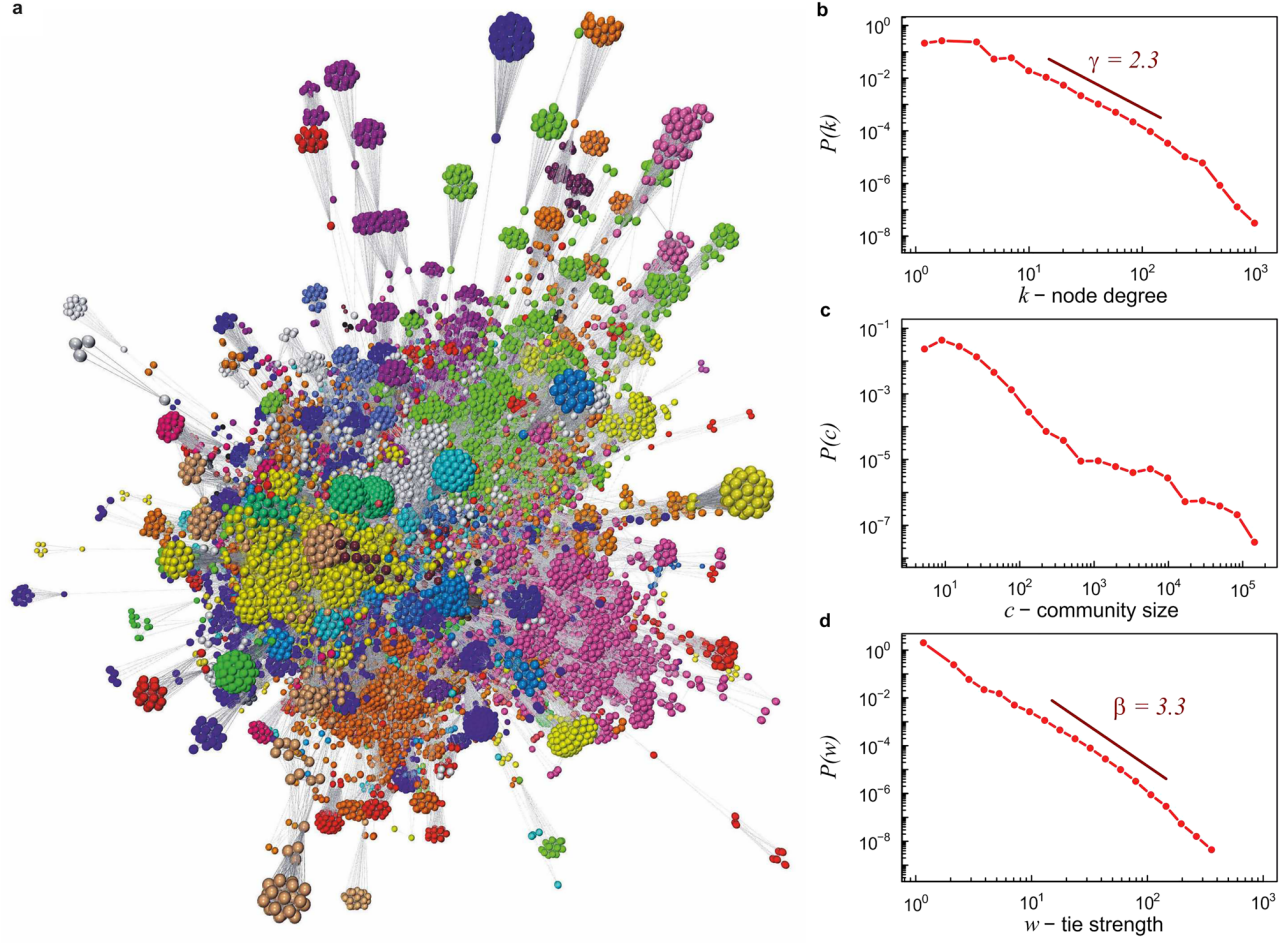
Basic structural properties of the real coauthorship network constructed using the DBLP computer science bibliography. (**a**) Visualization of the giant component of the network using Graphia application^28^. For better visibility only nodes with degree larger than 50 are shown resulting in the core network of almost seventy thousand nodes. The network is organized as a large number of communities. Each community was assigned a color according to the partitions identified by the Louvain algorithm^29^. (**b**, **c**, **d**) Logarithmically binned: node degree distribution *P*(*k*), community size distribution *P*(*c*), and link weight distribution *P*(*w*). The values $\gamma =2.3$ and $\beta =3.3$ shown in the graphs (**b**) and (**d**) correspond to the scaling exponents obtained by fitting power-law distributions to the relevant empirical data: $P(k)\propto k^{-\gamma }$ and $P(w)\propto w^{-\beta }$.

In the standard approach to scientific collaboration networks, the nodes represent authors, and an undirected internode connection occurs when two authors have published at least one paper together. When considered as binary networks—without any additional features assigned to nodes and connections—these networks show numerous structural similarities to other SNs (e.g. high clustering, small-world effect, skewed degree distribution and clear community structure; Fig. 1a,b,c)^14–17^. However, when edges are assigned weights representing, for example, the number of joint publications, then, although macroscopic characteristics of scientific collaboration networks (e.g., distributions of connection weights and node strengths; Fig. 1d) still correspond to those observed in typical SNs^7,26^, their microscopic structure related to the location of strong and weak ties is completely different. Dense, local neighbourhoods of nodes consist of weak ties, while strong ties act as bridges between local research groups. The atypical properties of scientific collaboration networks have been confirmed in several independent studies^9,11,27^.

**Figure 2:**
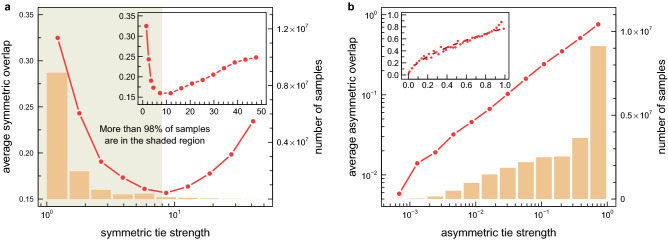
Dependence of neighbourhood overlap on tie strength in: (**a**) undirected, weighted DBLP scientific collaboration network, in which tie strength $w_{ij}$ corresponds to the number of joint publications (i.e. the number of times that co-authorship has been repeated) and the symmetric neighbourhood overlap $O_{ij}$ is given by the standard formula, Eq. (1); (**b**) directed, weighted projection of the same network with asymmetric tie strength $v_{ij}$ and asymmetric overlap $Q_{ij}$, obtained from Eqs. (3) and (2). In both graphs, circles indicate averages of overlaps (in intervals of logarithmically increasing width in the main panel and of constant width in the inset, respectively), while bars represent the number of samples from which the averages were calculated. Empirical relationships, similar to the one from the left graph (**a**), showing the decreasing character of $O_{ij}(w_{ij})$, have so far been the basic argument against validity of the Granovetter’s theory in scientific collaboration networks. The graph on the right (**b**) shows that the necessary condition to confirm the Granovetter’s theory in the studied networks is to reject the assumption about the symmetry of social ties.

Specifically, as shown in Ref.^11^, these unusual weight-topology correlations can be seen by analysing the relationship between the tie strength, $w_{ij}$, of two scientists *i* and *j*, and the overlap, $O_{ij}$, of their ego-networks. As indicated by Onnela et al.^7^, the overlap of two connected individuals is the ratio of the number of their common neighbours, $n_{ij}$, to the number of all their neighbours:

1 $$\begin{aligned} O_{ij}=\frac{n_{ij}}{(k_i-1)+(k_j-1)-n_{ij}}, \end{aligned}$$

where $k_i$ and $k_{j}$ represent degrees of the considered individuals. In typical SNs^30–33^, the above-defined overlap is an increasing function of the tie strength, $w_{ij}$, while analyses of scientific collaboration networks show something completely different. As can be seen in Fig. 2a, in the studied network of computer scientists, with $w_{ij}$ standing for the number of joint publications^34^, for the vast majority of connections ($98\%$), the overlap decreases with connection weight. This relationship indicates that weak ties mainly reside inside dense network neighbourhoods, whereas strong ties act as connectors between them. It has been hypothesized that this counterintuitive observation could be attributed to different driving mechanisms of tie formation and reinforcement in scientific collaboration networks in comparison to other social networks^11^. In what follows, we argue that the observation is related to the definitions of the tie strength and neighbourhood overlap that are not properly suited to the structure of the studied network.

**Figure 3:**
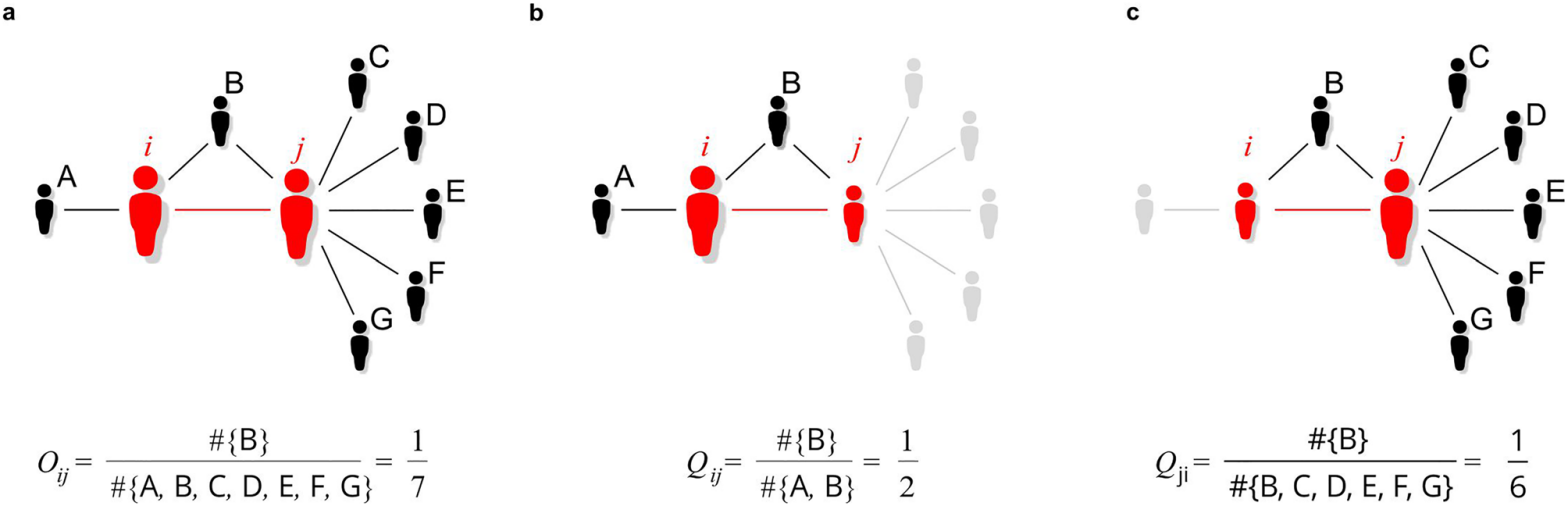
Illustration of the difference between symmetric and asymmetric neighbourhood overlap. In the figure, to highlight the benefits of analysing asymmetric overlaps, the corresponding values of: (**a**) symmetric $O_{ij}$ (1) and (**b**, **c**) asymmetric $Q_{ij}\ne Q_{ji}$ (2) overlaps have been calculated for the same network configuration, in which interconnected nodes differ in the size of their ego-networks. In such cases, which are typical for complex networks with underlying fat-tailed distributions, a common scenario is that for $k_i\ll k_j$ one has $Q_{ij}\gg Q_{ji}\simeq O_{ij}$. This explains why introducing tie direction is necessary for reliable verification of the Granowetter’s theory in scientific collaboration networks.

First, let us deal with the definition of the overlap (1) (referred to as *symmetric overlap*). In Fig. 3a, this local measure is shown in the case of a link connecting nodes with significantly different degrees. In such cases, for $k_i\ll k_j$, Eq. (1) can be simplified to $O_{ij}\simeq n_{ij}/k_j$, which shows that it is strongly biased towards nodes with high degrees, distorting the image of the common neighbourhood as seen from the perspective of nodes with small degrees. This drawback of symmetric overlap gains importance in networks with highly skewed, fat-tailed node degree distributions *P*(*k*). In such networks, as brilliantly exploited by the degree-based mean-field theory of complex networks^35–37^, node degree distributions for nearest neighbours are even more fat-tailed than the original distributions *P*(*k*). As a result, the number of edges in such networks connecting nodes with high and low degrees can be very high, leading to an unintended overrepresentation of strongly connected nodes by Eq. (1).

To overcome problems with symmetric overlap, we introduce the concept of *asymmetric overlap*:

2 $$\begin{aligned} Q_{ij}=\frac{n_{ij}}{k_i-1}\ne Q_{ji}. \end{aligned}$$

This can be used to describe the overlap between the neighbourhoods of two connected nodes from the perspective of each node separately. In the context of complex networks, this new definition is free from the shortcomings of the previous one. In particular, it copes well with connected nodes (collaborating scientists) whose degrees (ego-networks) differ significantly—that is, when their common neighbours (if any) are a significant part of the neighbourhood of one node and an insignificant part of the neighbourhood of the other. In such cases, the values of $Q_{ij}$ and $Q_{ji}$ corresponding to the same tie are different (see Fig. 3b,c). The value of $Q_{ij}$ that is close to 1 means that almost all neighbours of *i* are also neighbours of *j*. The value of $Q_{ji}$ close to 0 means that only a small part of the neighbourhood of *j* belongs to the neighbourhood of *i*.

The concept of asymmetric overlap naturally leads to the idea of directed networks and justifies the introduction of *asymmetric tie strength*:

3 $$\begin{aligned} v_{ij}=\frac{w_{ij}}{p_{i}}\ne v_{ji}, \end{aligned}$$

where $p_i$ stands for the number of all publications of the *i*-th scientist^38^. The intuitive rationale behind Eq. (3) is as follows: For a young scientist, with a small number of publications, each publication makes a significant contribution to his or her publication output, just as each co-author is an important part of his or her research environment (cf. Eqs. (2) and (3)). However, the importance of each publication and collaboration from the perspective of an established scientist with a large number of publications and an extensive network of collaborators is completely different. Depending on the circumstances, a given number of joint publications (e.g., $w_{ij}=1$) may have a completely different meaning.

In Fig. 2b, the dependence of asymmetric overlap on asymmetric tie strength for the considered network of computer scientists is shown. Contrary to what can be seen in Fig. 2a, the relationship $Q_{ij}(v_{ij})$ is increasing in the entire range of variability of its parameters. The result indicates that, from the point of view of a single scientist (ego-network approach), strong ties mainly constitute dense local clusters, whereas weak ties connect these clusters or play the role of intermediary ties^10^. The observation clearly confirms the validity of Granovetter’s first hypothesis in scientific collaboration networks.

Now, using the concept of asymmetric tie strength, we will discuss Granovetter’s second hypothesis, which postulates that although weak ties do not carry as much communication as strong ties do, they often act as bridges, providing novel, non-redundant information, which guarantees weakly connected nodes generally understood social success.

In scientific collaboration networks, the validity of Granovetter’s second hypothesis has never been tested. Nevertheless, it is widely believed (see^39^ and references therein) that information and expertise at the disposal of tightly connected research groups are often redundant, resulting in less creative collaborations and less innovative publications, while intergroup collaborations that bridge the so-called *structural holes*^40–42^ can provide access to information and resources beyond those available in densely connected communities, thus leading to novel ideas and valuable publications. To quantitatively address these issues, we check whether the bibliometric indexes of scientists and publications are correlated with the tie strength of the scientific collaboration network. Specifically, we focus on two questions: (i) How does the researcher’s h-index depend on the structure of his/her local collaboration network? (ii) How does the strength of the ties between scientists influence the success of their joint publication?

**Figure 4:**
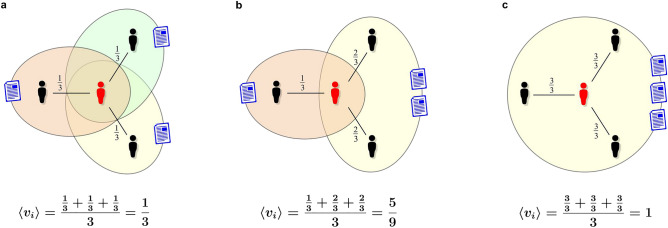
Average asymmetric tie strength of a scientist. The figure presents ego-networks of three different scientists (egos) with the same number of co-authors $k_i=3$ and publications $p_i=3$, but with different patterns of collaboration. On the left scheme (**a**), each of the three publications has only two authors; on the central scheme (**b**), two publications were written by a team of three and one by a team of two; in the scheme on the right (**c**), all publications involved the entire ego-network of a scientist. In each of the presented cases, the ego’s average asymmetric tie strength is different. Its value increases from the left diagram to the right, exactly in the same way as the intuitively understood social role of collaborators, on which depends not only the ego’s productivity but also integrity of his/her research group.

To answer the first question, we examined how the h-index^43,44^ of a scientist depends on his or her average asymmetric tie strength (see Fig. 4):

4 $$\begin{aligned} \langle v_i\rangle =\frac{1}{k_i}\sum _{j}v_{ij}. \end{aligned}$$

**Figure 5:**
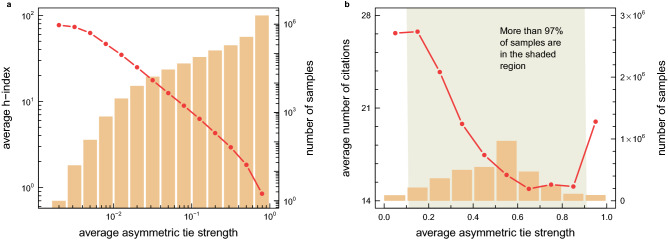
The role of tie configuration on scientific success of researchers and publications. (**a**) The mean h-index of scientists characterized by a given average asymmetric tie strength $\langle v_i\rangle$, Eq. (4). (**b**) The average number of citations obtained by papers created in teams with a given average asymmetric tie strength. The decreasing nature of both empirical relationships (**a**, **b**) clearly indicates that scientific collaboration based on weak ties is more appreciated in terms of the number of citations. Moreover, since the number of citations is often correlated with the quality of research, the above results also show that weak ties usually result in more creative (in terms of knowledge production) scientific collaborations.

**Figure 6:**
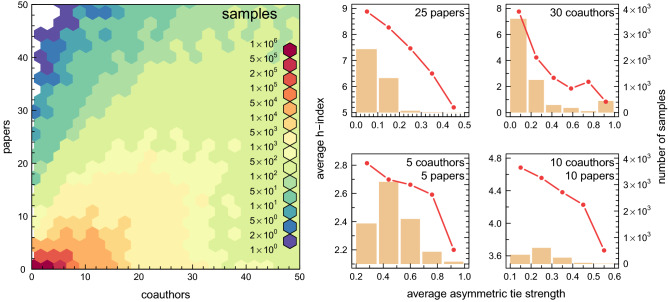
Scientists’ h-index versus tie strength. In this figure, we present a more detailed analysis of the relationship from Fig. 5a, which shows the data averaged over all scientists in the studied collaboration network, regardless of the stage of their scientific career. Here we divide scientists into groups in which everyone has the same number of total publications and the same number of co-authors (see the colour map in the figure). The more homogeneous conditions thus established allow us to clearly confirm earlier findings. In particular, as one can see in the small graphs on the right side of the colour map, regardless of the choice of the homogeneous group of scientists their h-index always decreases with increasing average asymmetric tie strength.

Equation (4) quantitatively measures the tendency of scientists to keep collaborating with the same people (cf. the concept of *social inertia*^45,46^). Figure 5a shows that the averaged (over all scientists who have a similar average tie strength) h-index decreases with $\langle v_i\rangle$. It means that successful (double-digit h-index) scientists have significantly weaker ties than less successful (single-digit h-index) researchers. The result is consistent with Granovetter’s general understanding of the role of weak and strong ties. However, since some doubts may arise from the fact that the data presented in Fig. 5a are averaged over many different scientists (having a small and large number of all publications, with a small and very extensive network of collaborators), in Fig. 6, we demonstrate that the decreasing nature of the relationship between the h-index and tie strength is independent of the choice of a group of scientists. That is, it still decreases, even in very homogeneous (in terms of scientific achievements) groups of researchers. In particular, as one can see in the small graphs accompanying the colour histogram that represents the available scientists’ samples, of any two researchers who have the same number of publications and/or co-authors, the one with weaker ties tends to have the higher h-index. In a way, this suggests that being a good manager and skilfully planning one’s network of scientific contacts ensures success^47^. This conclusion, however alarming as it may seem, finds its basis in the theory of social networks—the already mentioned concept of Burt’s structural holes and social capital^40,41^.

**Figure 7:**
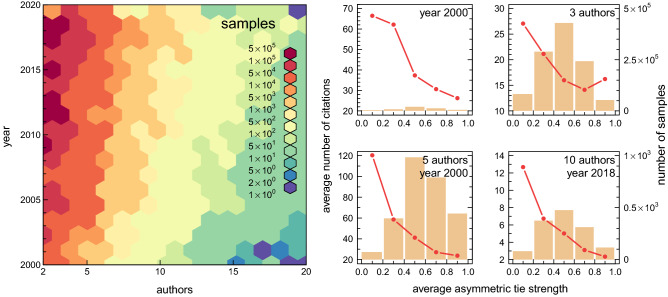
Citations of publications versus tie strength. In this figure, we present a more detailed analysis of the relationship from Fig. 5b. To this aim, all publications available in the analysed database are divided into groups according to the year of publication and the number of authors (see the colour map in the figure). Given homogeneous sample of publications thus established, we found that the number of their citations always decreases with increasing average asymmetric tie strength between their authors. To clarify, the average tie strength was determined at the time of paper’s publication, and the number of citations refers to the time of the last update of the analysed database.

The role of weak ties in scientific success is even more apparent in relation to scientific publications. Figure 5b shows how the number of citations of a scientific paper depends on the asymmetric tie strength (averaged over all co-authors of each article). The decreasing nature of this relationship indicates that publications created by teams of scientists linked by weak ties are better cited than those that arise in teams with strong ties. In Fig. 7, by analysing more homogeneous samples of publications (published in the same year and/or by the same number of co-authors), we clearly confirm the validity of the above finding. Furthermore, although the number of citations does not always translate into the quality of the research presented, it is undoubtedly a measure of the commercial success of a publication and a specific measure of the knowledge diffusion in scientific collaboration networks.

## Discussion and concluding remarks

The purpose of this work is to thoroughly verify Granovetter’s weak-tie theory of social networks. As clearly stated in the abstract and in the introduction: *Granovetter’s theory is based on two hypotheses that assign different roles to interpersonal, information-carrying connections*. Not all those who deal with the Granovetter’s theory pay attention to this distinction, which is undoubtedly crucial. The first hypothesis states that strong ties carrying the majority of interaction events usually correspond to intra-group connections. The second hypothesis maintains that weak inter-group ties, although less active, are of particular importance for the exchange of relevant information. A review of the literature reveals a striking disproportion between the research on the two hypotheses. In fact, the vast majority of empirical research to date has dealt with the first hypothesis, completely ignoring and sometimes not fully correctly interpreting the second one. In this respect our work is unique, because *we confirm Granovetter’s weak tie theory in its full spectrum*. And although in the absence of other studies, the analysis of the second hypothesis may seem to be the most important result of this work, our research on the verification of the first hypothesis also deserves attention as it highlights some important (and sometimes questionable or not entirely correct) threads in previous studies.

In particular, using massive datasets, clear empirical evidence for the first hypothesis, supported by the positive correlation between the symmetric overlap and tie strength, $O_{ij}(w_{ij})$, were reported in: mobile communication networks^7,30^, multiplayer online games^31,32^, and dialogues-based online SN^33^. On the other hand, the above mentioned methodology, exploiting symmetric network measures, failed in the analysis of scientific collaboration networks^9,11,27^, incorrectly classifying them as contradicting Granovetter’s theory. In this paper, we identify the reason why scientific collaboration networks behave differently than other SN. We argue that the U-shaped relation between $w_{ij}$ and $O_{ij}$ observed in coauthorship networks (see Fig. 2a) is related to the definitions of tie strength $w_{ij}$ and neighbourhood overlap $O_{ij}$ that are not properly suited to networks with scale-free node degree distributions. In any of the networks that were considered in Refs.^7,30–33^ this problem did not exist, because these networks were not truly scale-free (e.g. in mobile communication networks $P(k)\sim k^{-\gamma }$, with $\gamma =8.4$).

In this paper, to overcome the aforementioned issue, we have paid attention to the role of asymmetry in social ties. We have introduced new measures: asymmetric overlap $Q_{ij}$ and asymmetric tie strength $v_{ij}$, which not only allowed the successful verification of the first Granovetter’s hypothesis in scientific collaboration networks (see Fig. 2b), but have also opened the possibility to verify the second hypothesis. Moreover, as for the second hypothesis, which involves concepts related to the nature and importance of information, coauthorship networks have proved to be an extremely accurate choice, because: *(i) connections (ties) between network nodes (scientists) are well defined, and their weight (strength of ties) is easy to measure (e.g., through joint publications); (ii) scientific publications themselves are also a specific proxy of information flow in the studied network (diffusion of innovations); and (iii) the number of citations is an obvious measure of their significance.*

To be concrete, with regard to the second Granovetter’s hypothesis our results quantify what most scientists know very well: Scientific success is strongly correlated with the structure of a scientist’s collaboration network. We have explicitly shown that publications created by teams of scientists with weak ties are better cited than those that arise in teams with strong ties. And although this result was to be expected, it may be surprising that the differences in the number of citations of works created by weakly tied research groups compared to strongly tied groups amount not to a few or a dozen, but several hundred percent (see Fig. 7). Of course, when looking at these results quantitatively, one should bear in mind the limitations of the DBLP database used for the study. The database covers publications from computer science and includes publications from hybrid fields, where they are considered pertinent to computer science research. Papers from other disciplines are present there only occasionally. It means that super weak inter-domain ties are not covered by our analysis and the differences presented in Figs. 6 and 7 may be underestimated. On the other hand, computer science is quite heterogeneous due to the presence of many subfields, with very different norms in terms of team size and citation standards. Therefore, the results presented in Figs. 6 and 7 are aggregated over different subfields. Keeping above in mind, using more comprehensive database (e.g. Scopus or Web of Science), for the analysis reported in this study, can act as a double-edged sword. It would solve the first problem, but aggravate the second one. In this sense, our choice of the source of data seems to be a golden middle way.

Finally, an important research direction that was not undertaken in this paper, although it directly refers results reported here, is the issue of two recently discovered empirical scaling laws for social networks which relate link weight $w_{ij}$, symmetric overlap $O_{ij}$, and link betweenness centrality^48^ $b_{ij}$ in a non-linear way: $O_{ij}\propto \root 3 \of {w_{ij}}$ and $O_{ij}\propto 1/\sqrt{b_{ij}}$. Several studies (see e.g.^31–33^) have confirmed universality of these “social laws”. As we have already shown (cf. Fig. 2a and the corresponding figures in^9,11,27^), the first of these scaling laws—relating tie strength to the cube of the symmetric overlap—is not fulfilled in coauthorship networks. We have also checked that the same conclusion holds true for the second relation—expressing edge betweenness centrality as the inverse square of the overlap. In our case, the relationship $O_{ij}(b_{ij})$ is non-monotonic (non-increasing for small and intermediate values of betweenness and increasing for its large values, see Fig. S1 in Supplementary Information). Along these lines, we have also checked whether there is a clear correlation between tie strength and betweenness centrality and we have found no apparent dependency (see Fig. S2 in SI).

The additional analysis mentioned above provoke interesting research questions. The most controversial is whether the correlation between link betweenness centrality and symmetric overlap brings any relevant information about dynamical properties of social networks. In particular, whether the negative correlation between these measures provides quantitative evidence for the Granovetter’s theory. A kind of argument that supports these objections is that if we shuffle edge weights in a social network without changing the structure of its binary connections, then the weak ties hypothesis will surely cease to work, although the mentioned correlations will remain unchanged. Perhaps this argument could be refuted by using a kind of weighted/directed edge betweenness centrality, which, in combination with the asymmetric overlap $Q_{ij}$ introduced in this work, would allow for the formulation of more general laws of social dynamics than those formulated in^31^. An interesting way to overcome this problem has been proposed in^49^, where the authors pointed out that classical betweenness centrality is not useful to measure the influence of a team that is composed of more than two people^50^. Instead of this, a weighted hypergraph representation of the coauthorship network with higher-order interactions has been introduced and betweenness centrality measure has been adequately adapted to this new structure. In order to pursue studies on the role of weak ties in this direction, a new kind of overlap measure in hypergraphs has to be devised which itself seems to be challenging. The above considerations can be a starting point for interesting, new research on social networks.

## Data availability

The research presented in this paper is based on the publicly and freely available Citation Network Dataset^51^. We used the 12th version of the dataset (DBLP-Citation-network V12) which contains detailed information (i.e., year of publication, journal, number of citations, references, list of authors) and approximately 5 million articles published mostly during the last 20 years.

It is important to note that our analysis is limited to the largest connected component (LCC) in the co-authorship network, which can be recreated using the dataset. LCC comprises of close to three million nodes (authors), which means it spans 65% of the entire network. These nodes are connected by more than 13 million bi-directional co-authorship edges.

While the dataset provides exhaustive information about published papers, it does not directly contain any bibliometric information about authors. However, it is possible to calculate various bibliometric indicators either by recreating the network of citations or by directly using article metadata available in the dataset for each article (such as the number of citations). In order to calculate the h-index for all authors in the LCC, we decided to rely on the latter method and use article metadata to determine the number of citations. Considering that the citation network recreated from the dataset is only a sample of the full citation network, this method is more reliable. The number of citations calculated by counting links in the citation network is, in general, underestimated when compared with the number of citations available in the article’s metadata.

## Supplementary Information

Supplementary Information.

## Data Availability

The code that supports the findings of this study is available from the corresponding author upon request.
