# Supplementary material for: Scientific success from the perspective of the strength of weak ties
Source: Sci Rep. 2022 Mar 24;12:5074. doi: 10.1038/s41598-022-09118-8 (PMC8948253; doi:10.1038/s41598-022-09118-8)
Supplement: Supplementary file 1 — Supplementary Information. [file 41598_2022_9118_MOESM1_ESM.pdf]

Supplementary Material

for

"Scientific success from the perspective of the strength of weak ties"

Agata Fronczak, Maciej Mrowiński, and Piotr Fronczak

Below, we present the results of additional analysis of the studied network based on the edge betweenness centrality measure.

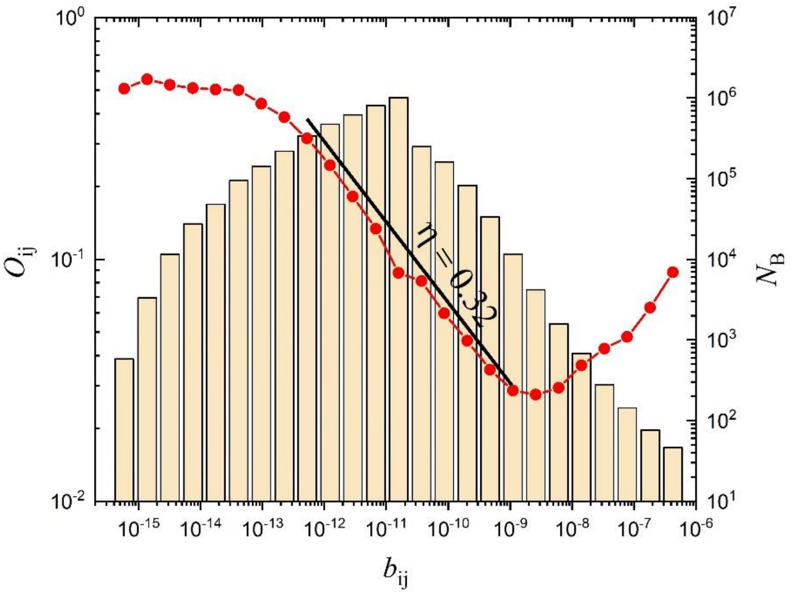

Fig. S1. Dependence of neighborhood overlap on edge betweenness in undirected DBLP coauthorship network. Circles indicate geometric averages of overlaps in intervals of logarithmically increasing width, while bars represent the number of samples from which the averages were calculated.

.

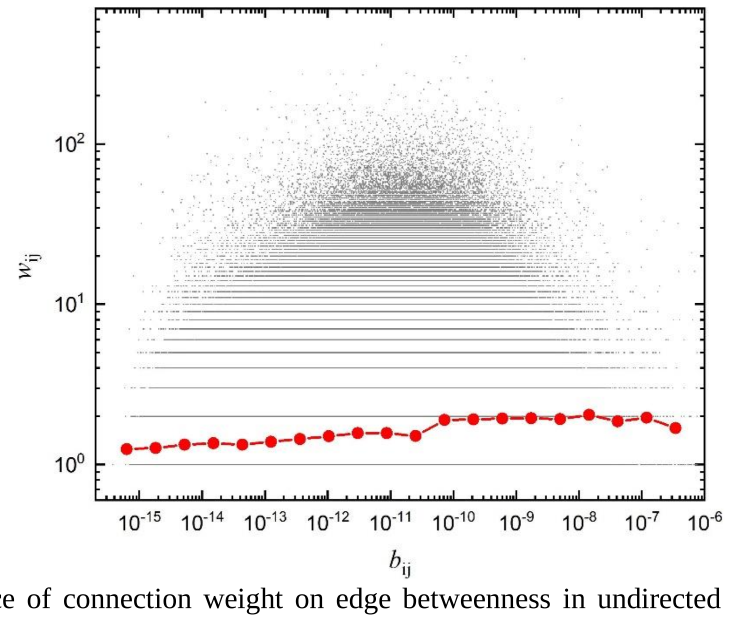

Fig. S2. Dependence of connection weight on edge betweenness in undirected DBLP coauthorship network. Circles indicate geometric averages of weights in intervals of logarithmically increasing width.
